# The incidence of multidrug and full class resistance in HIV-1 infected patients is decreasing over time (2001–2006) in Portugal

**DOI:** 10.1186/1742-4690-5-12

**Published:** 2008-02-01

**Authors:** Jurgen Vercauteren, Koen Deforche, Kristof Theys, Michiel Debruyne, Luis Miguel Duque, Susana Peres, Ana Patricia Carvalho, Kamal Mansinho, Anne-Mieke Vandamme, Ricardo Camacho

**Affiliations:** 1 Rega Institute, Katholieke Universiteit Leuven, Leuven, Belgium; 2 UCS. Dept. of Mathematics, Katholieke Universiteit Leuven, Heverlee, Belgium; 3 Hospital de S. Bernardo, Sétubal, Portugal; 4 Centro Hospitalar de Lisboa Occidental, Lisbon, Portugal; 5 Universidade Nova de Lisboa, Lisbon, Portugal

## Abstract

Despite improvements in HIV treatment, the prevalence of multidrug resistance and full class resistance is still reported to be increasing. However, to investigate whether current treatment strategies are still selecting for multidrug and full class resistance, the incidence, instead of the prevalence, is more informative. Temporal trends in multidrug resistance (MDR defined as at most 1 drug fully susceptible) and full class resistance (FCR defined as no drug in this class fully susceptible) in Portugal based on 3394 viral isolates genotyped from 2000 to 2006 were examined using the Rega 6.4.1 interpretation system. From July 2001 to July 2006 there was a significant decreasing trend of MDR with 5.7%, 5.2%, 3.8%, 3.4% and 2.7% for the consecutive years (P = 0.003). Multivariate analysis showed that for every consecutive year the odds of having a new MDR case decreased with 20% (P = 0.003). Furthermore, a decline was observed for NRTI- and PI-FCR (both P < 0.001), whereas for NNRTI-FCR a parabolic trend over time was seen (P < 0.001), with a maximum incidence in 2003–'04. Similar trends were obtained when scoring resistance for only one drug within a class or by using another interpretation system. In conclusion, the incidence of multidrug and full class resistance is decreasing over time in Portugal, with the exception of NNRTI full class resistance which showed an initial rise, but subsequently also a decline. This is most probably reflecting the changing drug prescription, the increasing efficiency of HAART and the improved management of HIV drug resistance. This work was presented in part at the Eighth International Congress on Drug Therapy in HIV Infection, Glasgow (UK), 12-16 November 2006 (PL5.5); and at the Fifth European HIV Drug Resistance Workshop, Cascais (Portugal), 28-30 March 2007 (Abstract 1).

## Background

In the last 2 decades, the management of HIV therapies has changed from the administration of one drug (monotherapy) to a combination of three or more antiretroviral drugs (HAART or Highly Active Anti-Retroviral Therapy). Currently, there are 24 single anti-HIV drugs approved by the FDA and new drugs are still getting developed. The potency of the current regimens is increasing and the drugs are getting better tolerated and easier to take. The mortality and morbidity of HIV infection has decreased in countries where therapy is available [1,2]. Changes in virologic response to initial combination antiretroviral therapy over calendar time indicate improvements in therapy [3], though it is too early to claim control of the infection on the long term. Therapy failure is due to such factors as lack of potency of the combination, insufficient drug adherence, and transmission of drug resistant virus [4], resulting in incomplete suppression of virus replication. Virus replication under drug selective pressure will invariably lead to increased drug resistance and cross-resistance, limiting further treatment options. Consequently, it is anticipated that drug resistance is and will continue to be a major issue in the effective treatment of HIV infection [5]. Especially, when HIV replication is not suppressed after exposure to several drug classes, multidrug resistance makes it difficult to optimize therapy and there is a higher incidence of AIDS and death [6,7]. Moreover, transmission of such multidrug resistant HIV is well documented [8], and is associated with suboptimal response to therapy [9], and (transmitted) resistance can persist over time [10,11].

Therefore, there exists an eagerness to identify new anti-HIV drugs active against resistant viruses, though data that quantify the problem of multidrug resistance (MDR) are rather confusing. The results seem even contradictory due to different settings of the studies performed and the way of defining MDR. Literature about the prevalence of resistance is numerous. Studies have shown that 5%–78% of treated patients harbor viruses resistant to members of two or more drug classes [12-15]. The wide range of values is mainly explained by the fact that some studies analyzed full class resistance (FCR) whereas other determined resistance to at least one antiretroviral drug in a given class. The use of different lists of mutations and/or different algorithms may also have played a role. Most papers report recent increases in prevalence of resistance [6,13,15-23], while few reports show a decrease of particular resistance profiles [13,22-25]. In the majority of these papers, the prevalence was calculated as the proportion of resistance in a certain time period which informs on the magnitude of the problem. Though this does not reveal the degree of newly acquired resistance, which can be checked by only considering the amount of resistance that was not yet detected before. The aim of this paper was to describe the trend in the incidence of multidrug and full class resistance over time, using definitions that are immediately relevant for the treating clinician. To our awareness, this is the first comprehensive longitudinal report on incidences of drug resistance over a long time period, 2001–2006, in a relatively stable epidemiological setting covering almost an entire country.

## Patients and Methods

**Figure 1:**
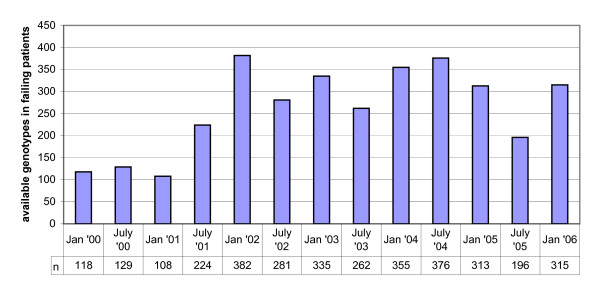
**Overview of available genotypes per half year in a Portuguese resistance database**. Since the implementation of routine resistance testing for treatment failure was constant from July 2001 onwards, the time frame between July 2001 and July 2006 was taken for trend analysis.

A Portuguese resistance database was used, containing genotypes of more than 4000 HIV-infected patients followed in 22 hospitals located over the whole Portuguese mainland and the Madeira Archipelago. Since January 2001, European guidelines recommend resistance testing in case of treatment failure [26]. From July 2001 to July 2006, the implementation of routine resistance testing for treatment failure was constant and the vast majority of samples were tested at Hospital Egas Moniz in Lisbon, the major reference laboratory. All available genotypes from treatment-experienced patients from 2000 onwards were included, however, to reduce as much as possible the effect of left censoring, only incidences between July 2001 and July 2006 were taken along in the statistical analysis. Thus, patients with samples that showed MDR or FCR before July 2001, were excluded from the incidence analysis of MDR or FCR, respectively. See Figure 1.

The genetic data resulted from population sequencing using Trugene HIV-1 genotyping (Bayer Diagnostics) in 2000 and first half of 2001 and an automated sequencer (ABI Prism 3100, Applied Biosystems) plus a commercially available assay (ViroSeq HIV-1 Genotyping System, v2.0, Abbott) from 2001 onwards. Sequences that did not cover the full region of HIV-1 RT and HIV-1 PRO related to resistance were excluded. The genotypic results were interpreted by using the Rega algorithm (version 6.4.1) [27,28] for the following drugs: zidovudine, didanosine, lamivudine, stavudine, abacavir, emtricitabine, tenofovir, nevirapine, delavirdine, efavirenz, saquinavir, indinavir, nelfinavir, amprenavir, lopinavir (boosted-), atazanavir and atazanavir (boosted-). The algorithm scores genotypes as susceptible, intermediate resistant and resistant; for the purposes of this study, intermediate resistant and resistant were considered as "resistance". When the algorithm scored viruses as "susceptible" to at most one of these drugs, the patient was assumed to harbor a multidrug resistant virus (MDR) reflecting the difficulty to select an efficient HAART regimen. Because tipranavir and darunavir were licensed only in June 2005 and 2006, respectively, these drugs were not available at all time points of the study, so resistance or susceptibility to these drugs was not considered. Concomitantly, as zalcitabine is no longer used in Portugal, resistance to it was neither considered. Additionally, because administration of the fusion inhibitor enfuvirtide was approved only in mid-2003 and resistance testing for this drug is rarely undertaken, drug-resistance to it was not addressed. Resistance to ritonavir was not taken into account since this drug is only administrated in order to boost other protease inhibitors. Intermediate or high level resistance to all drugs in a class lead to the establishment of full class resistance (FCR). Analyses were repeated by using Stanford HIVDB (version 4.1.9) and ANRS (version 2005.07) interpretation systems. The three studied classes are nucleoside reverse-transcriptase inhibitors (NRTIs), nonnucleoside reverse-transcriptase inhibitors (NNRTIs), and protease inhibitors (PIs).

The data were grouped into consecutive periods of 12 months: from July of a year to June of the next year, since data were only representative after July 2001 as mentioned above, and inclusion was up to July 2006, in order to achieve representative subpopulations with a sufficient power (Figure 1). For a given time period, the incidence of resistance was defined as the proportion of patients in which a resistant virus was detected for the first time with respect to the total number of patients with a resistance test in that time period. If resistance is detected in a patient's virus for the first time, then all of his follow-up samples were excluded from the analysis, thus preventing the cumulative effect of resistance and thus calculating incidence and not prevalence. Analyses were repeated by using information on total number of treated patients in Portugal based on information on medical prescription and collected from hospital pharmacies.

The data were analyzed using the free statistical software R (version 2.3.1). The statistical analysis consisted of three steps. In a first preliminary stage, trends in incidences over time were investigated by the Chi-squared based test for trend in proportions. Secondly, the incidences were modeled over time using a (univariate) Poisson regression model and graphically visualized. Factors that could bias the results were the time elapsed since a patient started therapy and the genotyping, and the fact that for some patients the date of therapy initiation was not exactly known. Therefore, in a third and final stage, trends over time were corrected for confounding factors using multivariate logistic regression. 95% confidence intervals (95%–CI) were calculated based on the binomial and the normal distribution.

To verify whether the trends in resistance in treated patients are reflected in trends in transmission of drug-resistant virus, temporal trends of resistance in newly diagnosed treatment naïve patients in Portugal were examined by using data prospectively collected from 2003 to 2005 as part of the pan-European SPREAD program [29,30].

## Results

A total of 2702 therapy-experienced patients were identified in a Portuguese database as having at least one genotypic resistance test between January 3, 2000 and June 30, 2006. In total 3394 sequences were scored using the Rega 6.4.1 algorithm. Since implementation of routine resistance testing for treatment failure was constant only from July 2001 onwards, and in order to reduce the effect of left censoring, incidence figures only for the period July 2001 to July 2006 were taken into account. If the patient's virus does not show resistance at a first time point, then possible consecutive sequences can be included for the other time-periods. But if the sequence is scored as drug resistant, then all other subsequent sequences are excluded from the analysis. This results in a change in the denominator in consecutive years. Table 1 provides the number and incidence of observed resistance over time in this cohort of patients.

**Table 1:** Incidences and Chi-squared based test for temporal trends in resistance

		2001–'02	2002–'03	2003–'04	2004–'05	2005–'06	P-value
**Multidrug resistance**							
	n	33	30	22	21	13	
	N	576	574	583	626	490	
	incidence	5.7	5.2	3.8	3.4	2.7	0.003
	95%-CI	4.0 – 8.0	3.5 – 7.4	2.4 – 5.7	2.1 – 5.1	1.4 – 4.5	
**Full class resistance**							
NRTI	n	78	63	53	40	29	
	N	576	572	578	619	476	
	incidence	13.5	11,0	9.2	6.5	6.1	<0.001
	95%-CI	10.9 – 16.6	8.6 – 13.8	6.9 – 11.8	4.7 – 8.7	4.1 – 8.6	
NNRTI	n	203	226	258	245	187	
	N	570	557	541	560	445	
	incidence	35.6	40.6	47.7	43.8	42.0	0.011
	95%-CI	31.7 – 39.7	36.5 – 44.8	43.4 – 52.0	39.6 – 48.0	37.4 – 46.8	
PI	n	60	50	32	40	35	
	N	574	570	572	611	479	
	incidence	10.5	8.8	5.6	6.5	7.3	0.013
	95%-CI	8.1 – 13.2	6.6 – 11.4	3.9 – 7.8	4.7 – 8.8	5.1 – 10.0	

NRTI, nucleoside reverse-transcriptase inhibitors; NNRTI, nonnucleoside reverse-transcriptase inhibitors; PI, protease inhibitors.

Multidrug resistance (MDR): at most 1 susceptible drug available over all classes; Full class resistance (FCR): no susceptible drugs available in a class.

**Figure 2:**
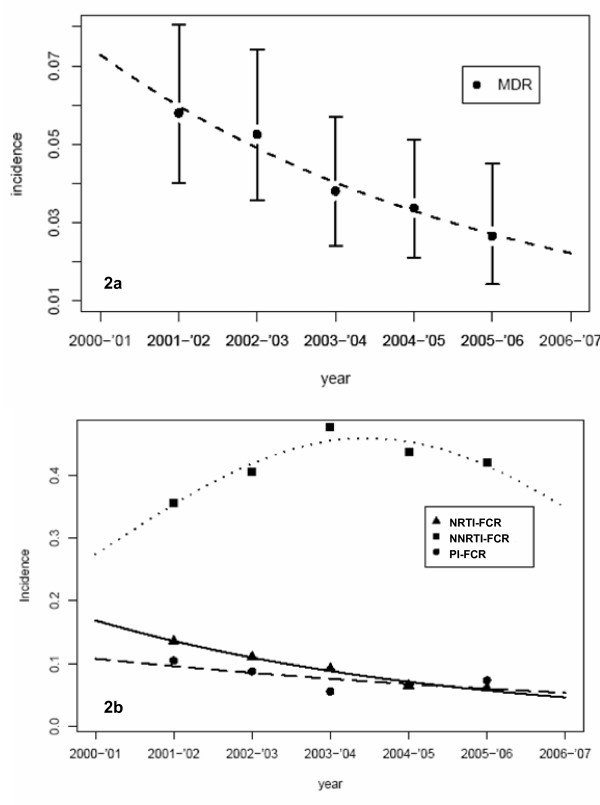
**Incidence of resistance over time (2001–'06) based on data of a Portuguese resistance database**. 2a: Multidrug resistance (MDR): at most 1 susceptible drug available over all classes. A Poisson regression model was fitted on the data (trend line) that showed a significant decreasing trend (OR = 0.82, 95%-CI: 0.72 – 0.94; P = 0.004). 2b: NRTI-, NNRTI- and PI- full class resistance (FCR): no susceptible drugs in that class available. Significant Poisson regression models were fitted on the data (trend lines).

The incidence of patients harboring a virus with MDR continuously decreased in Portugal between 2001 and 2006. From July 2001 to June 2002, 33 out of 576 (5.7%) patients carried a virus which was estimated to be susceptible to not more than 1 drug, whereas in the time between July 2005 and June 2006 this proportion of new cases of MDR fell to 13 out of 490 (2.7%) patients. Table 1 also holds a P-value of 0.003 for the preliminary Chi-square based statistical test for trends in proportions. The significance of the trend was confirmed by using (univariate) Poisson regression, a model that is widely used to study event count data (P = 0.004). The incidences of MDR are plotted on Figure 2a together with the fit of the Poisson model (dashed trend line). Two possible confounding factors were investigated. On the one hand, the on-therapy time when the sample for resistance testing was taken, can influence the incidence of resistance. On the other hand, for 45% of the patients the on-therapy time could be underestimated since the date of therapy initiation was not certain. To rule out these potential biases, a multivariate logistic regression model was used in which the significant time trend was confirmed: for every consecutive year the odds of having a new MDR case decreased by 20% (OR = 0.80, 95%-CI: 0.69 – 0.93; P = 0.003). For every extra year on therapy, the odds of evolving to a multidrug-resistant virus increased by 16% (OR = 1.16, 95%-CI: 1.09 – 1.23; P < 0.001). Patients with no certain start date of therapy were almost 8 times more likely to develop a MDR virus (OR = 7.88, 95%-CI: 4.60 – 13.48; P < 0.001) which may reflect the fact that these patients started therapy before 1998, when therapy initiation records were finally kept on digital format.

The same steps of statistical analysis were performed for different definitions of resistance. For FCR (full class resistance: no susceptible drugs available anymore in a class), all incidences are shown in Table 1. The trends over time and the fitted Poisson models are shown in Figure 2b (for NRTI-, NNRTI- and PI-FCR). For all classes, previous time on therapy was unmistakably associated with resistance. Multivariate analysis demonstrated that NRTI-FCR is clearly decreasing over time: from 13.5% in 2001–'02 to 6.1% in 2005–'06. For every consecutive year the odds of harboring a NRTI-FCR virus decreased with 26% (OR = 0.74, 95%-CI: 0.70 – 0.82; P < 0.001). In contrast, the incidence of patients carrying a virus resistant to all NNRTIs increased from 2001–'02 onwards (35.6%), reaching a maximum in 2003–'04 (47.7%), but then decreased again (42.0% in 2005–'06). This parabolic trend was statistically validated (P < 0.001). For PIs, an overall decrease in resistance was found to be statistically significant (OR = 0.80, 95%-CI: 0.71 – 0.89; P < 0.001), though interestingly, the incidence started at 10.5% in 2001–'02 but then came to its lowest level in 2003–'04 (5.6%) and then rose again in the following 2 years (7.3% in 2005–'06). This parabolic trend was only borderline significant (P = 0.106).

There have been many different ways of looking at trends in resistance. Throughout the literature, various definitions of resistance are used, all of which were applied to our data. To avoid confusion, only a few of those additional results are briefly summarized here. All incidence trends were confirmed by using Stanford HIVDB (version 4.1.9) or ANRS (version 2005.07) as interpretation algorithm. When scoring resistance as at least one drug in a class that is associated with reduced susceptibility, similar results as FCR were obtained: linear decreasing time trends for NRTI-resistance (OR = 0.83, 95%-CI: 0.77 – 0.90; P < 0.001) and PI-resistance (OR = 0.67, 95%-CI: 0.63 – 0.72; P < 0.001) and parabolic (up and down) time trend for NNRTI-resistance (P < 0.004). When analyzing trends in incidence of resistance with respect to an estimation of all patients in Portugal on therapy, the same trends are again confirmed, with even higher support (all P < 0.001). Finally, analysis of resistance data in drug-naïve patients collected in Portugal from 2003 to 2005 showed a declining trend in NRTI-resistance and a up-and-down trend in NNRTI-resistance, though not significant most probably due to lack of statistical power [30]. Baseline resistance to PIs was so rare that no time trend could be studied.

## Discussion

One of the most pressing questions for clinicians to date is whether the problem of multidrug resistance (MDR) and full class resistance (FCR) is related mainly to inefficient regimens and management of HIV drug resistance in the past, or whether current treatment strategies are still selecting for MDR and FCR. This information is also of great importance for drug developers since they need to know where the biggest challenges lie: developing potent antiretroviral agents targeted at overcoming resistance or focusing more on better tolerability and ease of use of the drug. To answer these questions, it is better to examine the incidence of resistance. However, previous attempts to estimate the extent of multidrug resistance have generally used prevalence and not incidence as measurement unit. The prevalence gives the ratio (for a given time period) of the number of occurrences of resistance to the number of units at risk in the population, whereas the incidence stands for new occurrences. Prevalence data are also important because they can give an idea of the magnitude of the problem of resistance which is crucial knowledge because individuals with resistance may transmit these viruses to others [31], and because these patients need to find an effective treatment at the time of measurement. Moreover, the requirement for new classes of drugs for those patients infected with multidrug resistant virus must be quantified. As discussed in the introduction, most publications state that the prevalence of resistance is increasing over time, resulting in the continued focus of drug designers on the activity of drugs against resistant viruses [32,33]. However, since prevalence statistics are cumulative, these do not reflect trends on newly acquired resistance. Therefore, for the purpose of this study, incidence is a more appropriate statistic to use.

In this paper, time trends of drug resistance incidence were investigated by using clinically relevant definitions. 'Multidrug resistance' (MDR) was defined as at most one fully active drug reflecting the difficulty to install an effective treatment. 'Full class resistance' (FCR) was defined as no drug in that class that is fully active reflecting the loss of an entire class of drugs. The incidence was defined as the proportion of the number of patients with an incident resistant virus with respect to the total number of treated and genotypically tested patients. From 2001 to 2006, a significant declining trend of MDR incidence was noticed. Multivariate analysis showed that for every consecutive year the odds of having a new MDR case decreased with 20%. Furthermore, a decline was observed for NRTI- and PI-FCR, whereas NNRTI-FCR showed an up-and-down trend over time. These overall declining trends of resistance in treated patients may lead to reduced transmission of drug-resistant virus. To verify this assumption, the results were compared to temporal trends of resistance in untreated patients. For resistance to NRTIs and NNRTIs the trends were similar as in treated patients: downwards and up-and-downwards respectively. Baseline resistance to PIs was so rare that no relevant conclusions could be drawn. Nevertheless, it should be noted that resistance can also be transmitted by other drug naïve HIV-infected individuals [34,35], so that baseline resistance doesn't necessarily follow directly the trends of resistance in treated patients.

As mentioned in the introduction, in literature there are several ways of looking at resistance, though we believe that our way of defining MDR (as at most 1 drug over all classes still susceptible) is clinically the most relevant one since it reflects the situation in which it is hard to optimize treatment. Nevertheless, additional analyses were performed showing that with other commonly used definitions of resistance, the same trends were seen. A significantly declining trend was found for 'any' resistance (resistance to at least 1 drug), for triple FCR (no susceptible drugs in any class) and for resistance to at least 1 drug in every class. Furthermore, all trends were confirmed by using Stanford HIVDB (version 4.1.9) or ANRS (version 2005.07) in stead of Rega 6.4.1. For the clarity of this paper, these results are not shown.

It can be argued that at the beginning of our study period, resistance testing started to be implemented in routine clinical practice such that relatively more advanced and drug resistance patients were tested for the first time (left censored bias). To cope with this problem, patients with samples that showed MDR, or FCR before July 2001, were excluded from the analysis of MDR, or FCR respectively. While this may not have eliminated the bias entirely, the continuing decreasing trends of MDR (and FCR) even in the most recent years suggests that these are genuine.

The study population is representative for all patients failing on therapy in Portugal, since the data cover 22 hospitals located over the entire country, since from July 2001 onwards it is routine to perform resistance testing for every virologically failing patient and since the majority was tested at Hospital Egas Moniz. In fact, the results for MDR could even be extrapolated to the whole treated population in Portugal, since every patient on treatment is normally genotypically tested when failing. Our analysis was performed using the number of patients with a genotype in the denominator for the incidence, since these are the failing patients in which resistance can be expected. When analyzing the incidence with respect to all patients on therapy, the trends are confirmed. However, these results are less reliable since there is no official body in Portugal collecting such data; the total number of patients on therapy was derived from information based on prescription behavior and collected from hospital pharmacies.

We have shown that the incidence of MDR declined over time in Portugal. Furthermore, we found some other interesting changes in the degree of resistance over the studied time period. NRTI-FCR decreased significantly over time which parallels the more potent NRTIs being administrated and possibly the protective role of the other drugs in the regimen (e.g., boosted protease inhibitors). The rising prescriptions of NNRTIs during the late nineties and early years of 2000 enhanced the occurrence of NNRTI associated mutations. However, since NNRTI prescription stabilizes and since the NRTI backbone (more tenofovir + emtricitabine instead of zidovudine + lamivudine) possibly becomes more potent and tolerable [36], thus enhancing adherence, the NNRTI resistance seems to lower again. This might explain the significant parabolic trend (up and down) for NNRTI-FCR over time. An overall declining tendency is observed for resistance to PIs, though the PI-FCR decline seems to level off. This parabolic time trend was however only borderline statistically significant. The linearly decreasing temporal trend of MDR and of FCR (except for NNRTIs) reflects the more successful suppression of the virus replication by modern HAART. This can be attributed to the recent more potent drug combinations, which continue to improve, the better clinical management of the disease, the improved adherence, the extended experience of the clinician and the assistance of the virologist through the interpretation of the genotypic resistance information. In particular, the decline in resistance can be partially explained by the fact that in the early years of 2000, treatment initiation in patients was delayed (less 'hit early, hit hard'); so as a consequence, these patients were subsequently treated with newer and better drug combinations. As the incidences also decrease in more recent years and since HAART keeps on improving, a new rise is not expected.

Even though we do not want to extrapolate our findings, the same declining trends of resistance are expected in other European countries. Nevertheless, caution is needed, since only prevalences and not incidences reveal how many patients are currently struggling with resistance and they have to be taken care of. Resistance is and will continue to be a major concern in the management of HIV infection. Clearly, new drugs targeting other steps in HIV-1 replication, and with no cross-resistance to previous classes of antiretrovirals, are a very important advance for the clinical management of drug-experienced patients. Recent developed drugs such as etravirine, raltegravir, maraviroc, darunavir, tipranavir and enfuvirtide will improve the outcome of a significant number of patients. The analyses were done without taking these drugs into account since they were not available at all time points of the study. Furthermore, data on other factors that might influence the incidence of resistance such as the exact number of days on therapy, persistence of resistance, adherence information and the higher impact of adherence on resistance to NNRTIs than to PIs [37], number of patients on first, second, or more advanced lines of therapy, etc. were not available.

## Conclusion

Our data indicate that the incidence of multidrug-resistant HIV-1 is decreasing over time in Portugal, reflecting the increasingly efficient management of treatment and resistance. To our knowledge our study is the first to use incidence figures of resistance for modeling the trend of acquiring resistance over time, in a relatively stable epidemiological setting covering almost an entire country. With our data, we predict that new drugs, active against multidrug resistant virus, without improved toxicity, tolerability and half life compared to existing drugs, may be beneficial only for a limited and declining number of multidrug resistant patients. In the development of new drugs, it may therefore be more, or certainly equally, important to improve features that are relevant for drug adherence – like a better tolerability, ease of use or less toxicity – such that a larger number of patients will profit. By improving adherence, the use of such improved drugs will presumably decrease even further the incidence of resistance.

## Competing interests

All authors received travel grants from the pharmaceutical industry. Kamal Mansinho, Anne-Mieke Vandamme and Ricardo Camacho participated in advisory boards for several pharmaceutical companies. The funding sources had no role in study design; collection, analysis, or interpretation of data; or in the writing of the report.

## Authors' contributions

JV performed all analyses and wrote the manuscript. KD and KT collected and sorted the data relevant for this study. MD supported the statistical analyses. LMD, SP and APC have collected the samples, performed resistance testing and contributed to the interpretation of the resistance results. KM coordinated the collection of clinical data. The study was designed and supervised by A-MV and RC. All authors have participated in the discussion of the results and writing of the manuscript.
